# Supplementary material for: Geospatial analysis of leptospirosis clusters and risk factors in two provinces of the Dominican Republic
Source: PLoS Negl Trop Dis. 2025 Jun 11;19(6):e0013103. doi: 10.1371/journal.pntd.0013103 (PMC12157080; doi:10.1371/journal.pntd.0013103)
Supplement: S1 Text — (DOCX) [file pntd.0013103.s001.docx]

**Supporting Information**

**Geospatial analysis of leptospirosis clusters and risk factors in two provinces of the Dominican Republic**

1. **Study design and participant selection:**

The analysis presented in our study is part of a larger research project entitled “Defining the pathogens, transmission characteristics, risk factors and geospatial hotspots of acute febrile infections (AFIs) in the Dominican Republic (DR). This research project had two arms, (i) a prospective AFI surveillance study conducted at two sub-national hospitals located in Espaillat and San Pedro de Macoris between 2019 and 2023, and (ii) a national, population-representative cross-sectional serological survey conducted between June and October 2021. In our study, we used data obtained in the survey arm. The full description of the study design was previously reported by Nilles, et al, 2022 (1). Results for leptospirosis seroprevalence were available only for participants from the two provinces linked with the prospective AFI surveillance. A full description of the surveillance study design can be found in Nilles et al, 2023 (2).

**References:**

1. Nilles EJ, Paulino CT, De St. Aubin M, Restrepo AC, Mayfield H, Dumas D, et al. SARS-CoV-2 seroprevalence, cumulative infections, and immunity to symptomatic infection – A multistage national household survey and modelling study, Dominican Republic, June–October 2021. The Lancet Regional Health - Americas. 2022;16:100390.

2. Nilles E, de St. Aubin M, Dumas D, Duke W, Etienne MC, Abdalla G, et al. Monitoring Temporal Changes in SARS-CoV-2 Spike Antibody Levels and Variant-Specific Risk for Infection, Dominican Republic, March 2021–August 2022. Emerging Infectious Disease journal. 2023;29(4):723.
