# Supplementary figures and images for: Geospatial analysis of leptospirosis clusters and risk factors in two provinces of the Dominican Republic
Source: PLoS Negl Trop Dis. 2025 Jun 11;19(6):e0013103. doi: 10.1371/journal.pntd.0013103 (PMC12157080; doi:10.1371/journal.pntd.0013103)

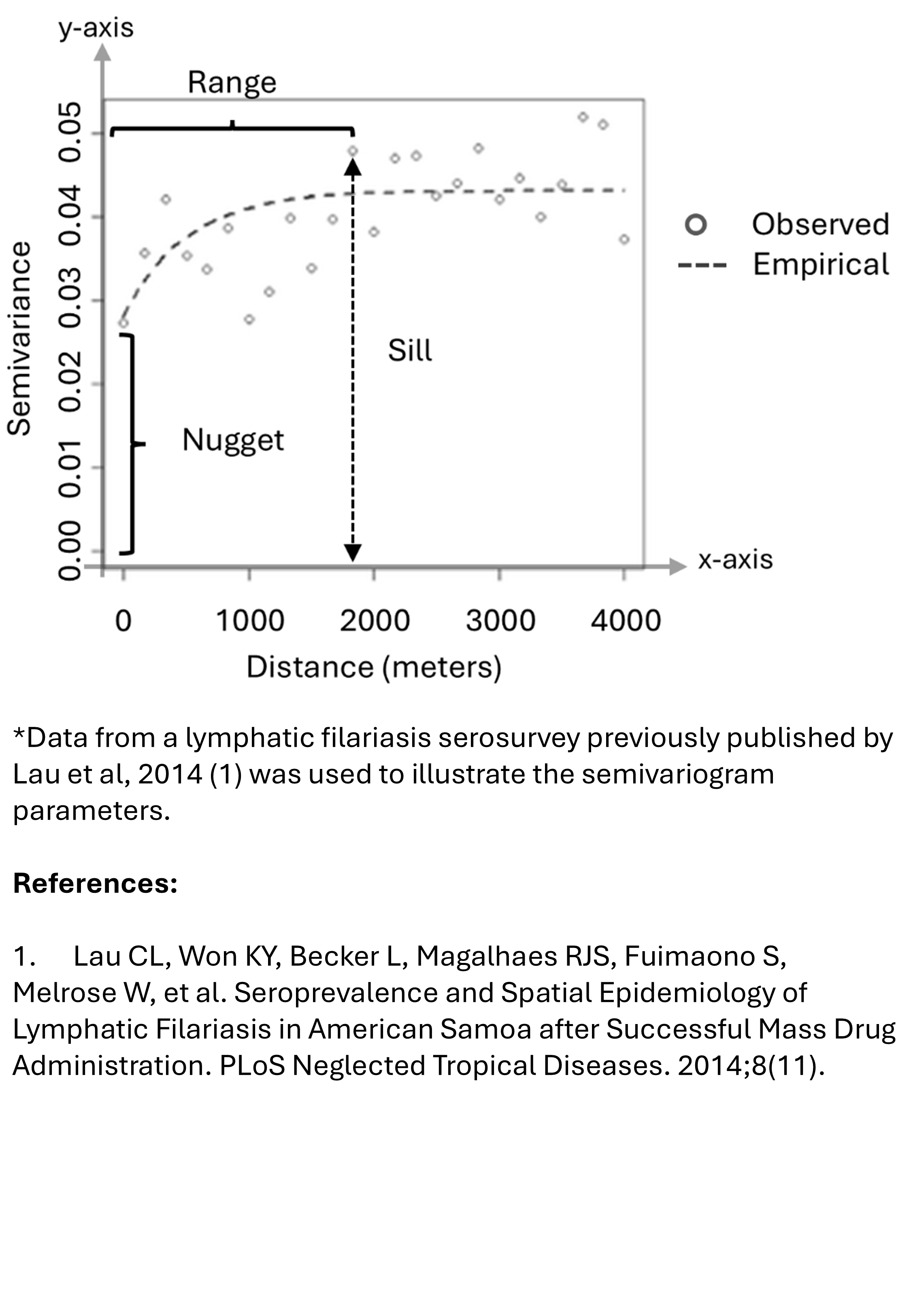

Supplement: S1 Fig — Data from a lymphatic filariasis serosurvey previously published by Lau et al, 2014 [55], was used to illustrate the semivariogram parameters. (TIF) [file pntd.0013103.s004.tif]

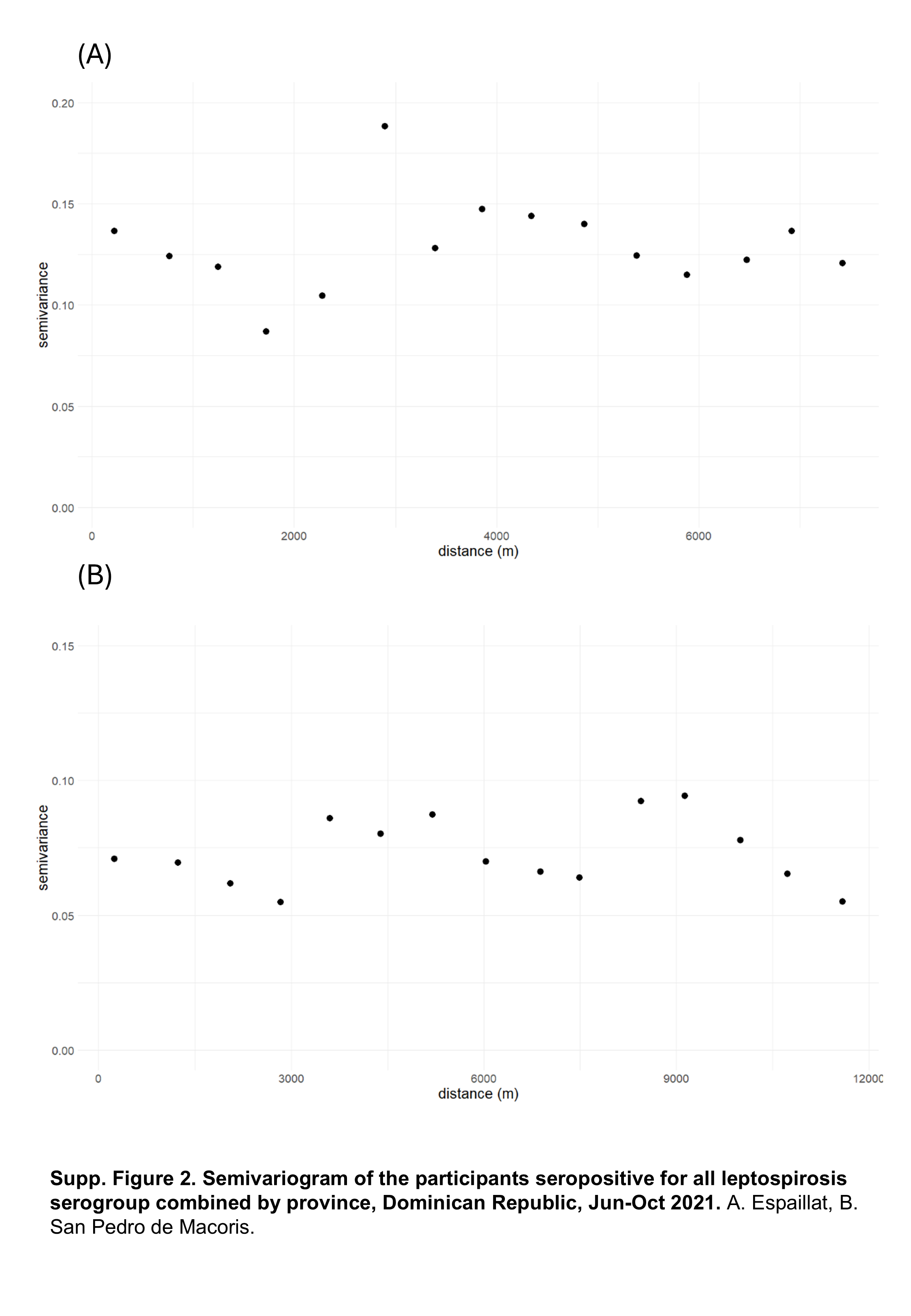

Supplement: S2 Fig — A. Espaillat, B. San Pedro de Macoris. (TIF) [file pntd.0013103.s005.tif]

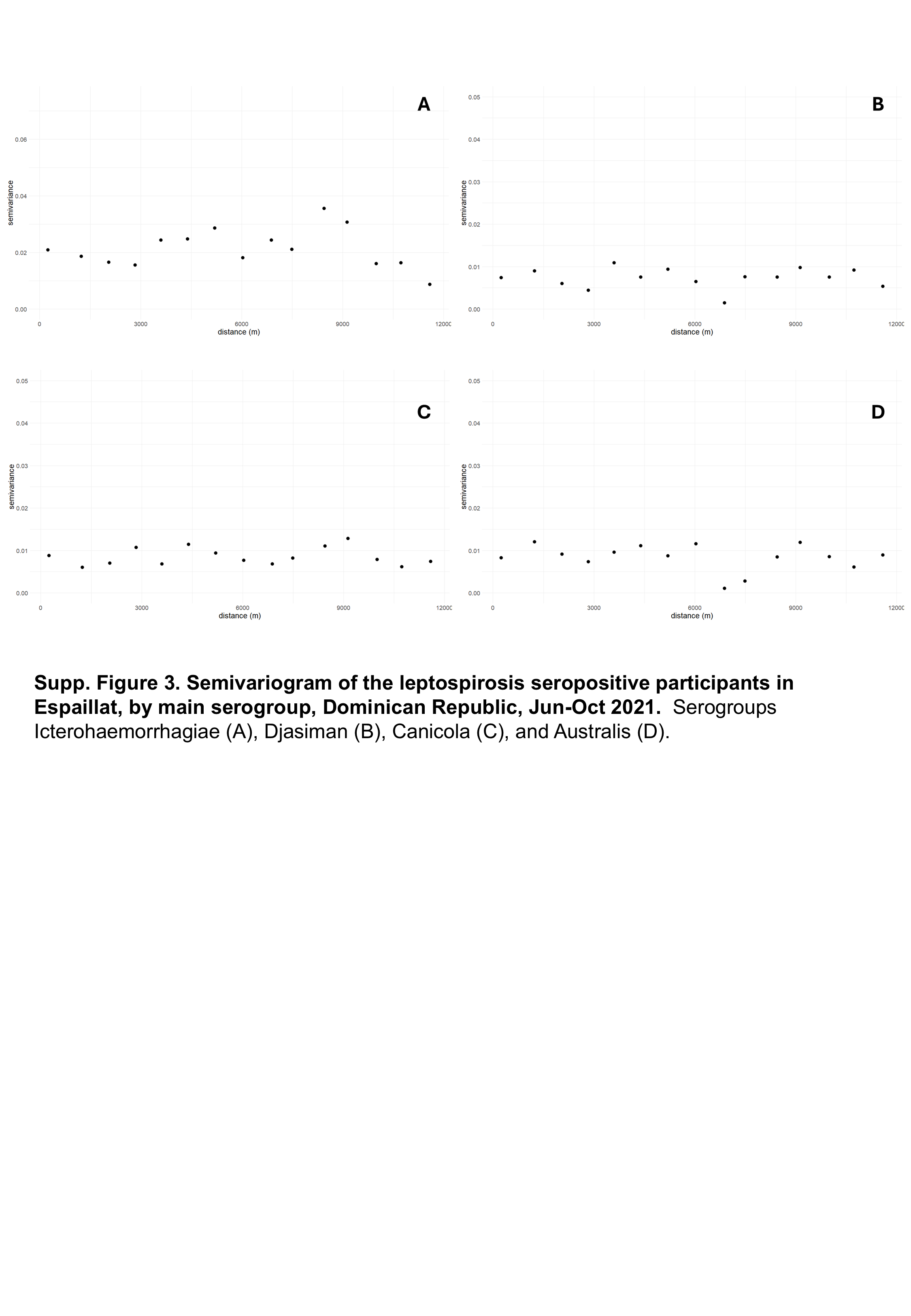

Supplement: S3 Fig — Serogroups A. Icterohaemorrhagiae B. Djasiman C. Canicola D. and Australis. (TIF) [file pntd.0013103.s006.tif]

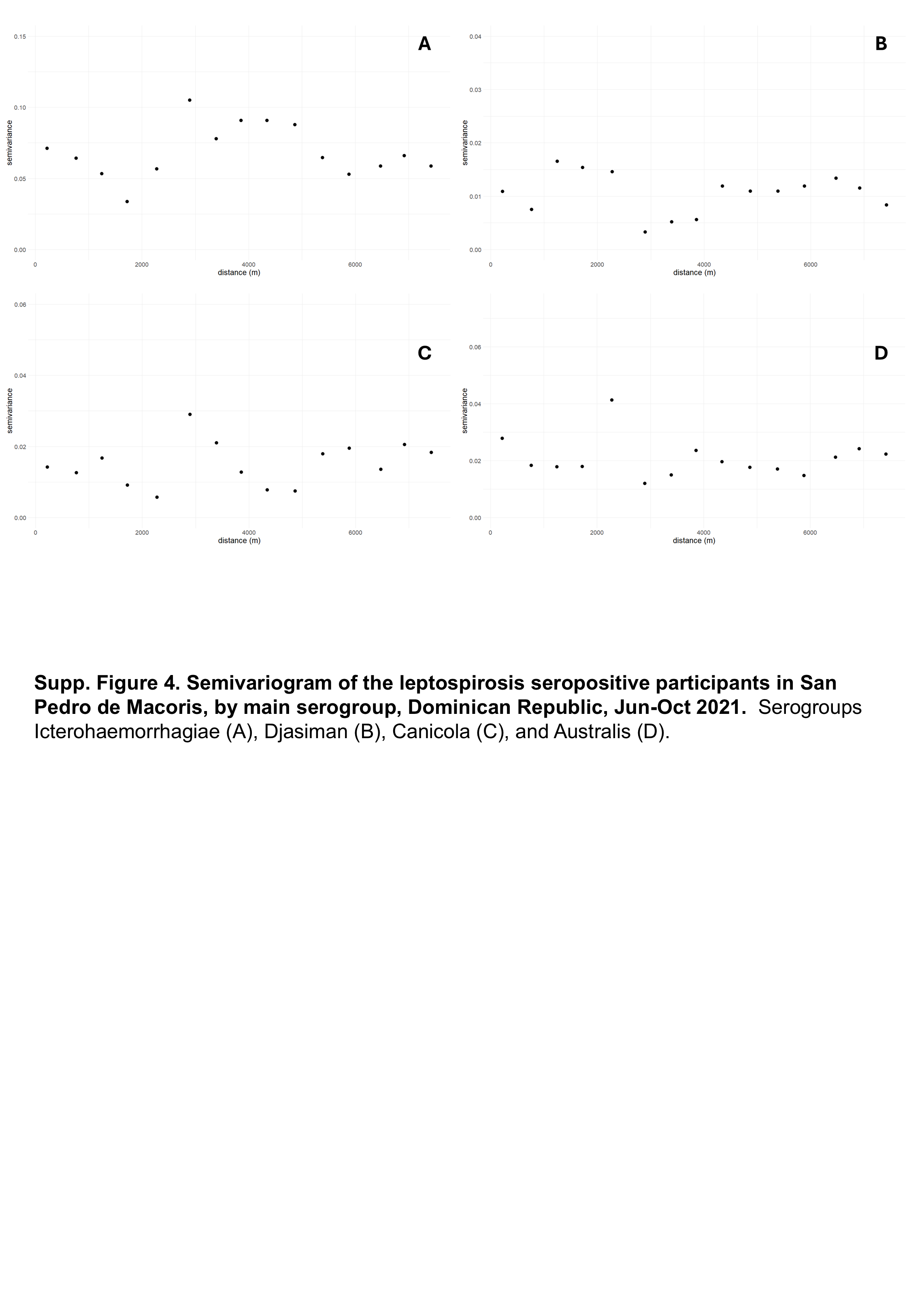

Supplement: S4 Fig — Serogroups A. Icterohaemorrhagiae B. Djasiman C. Canicola D. and Australis. (TIF) [file pntd.0013103.s007.tif]

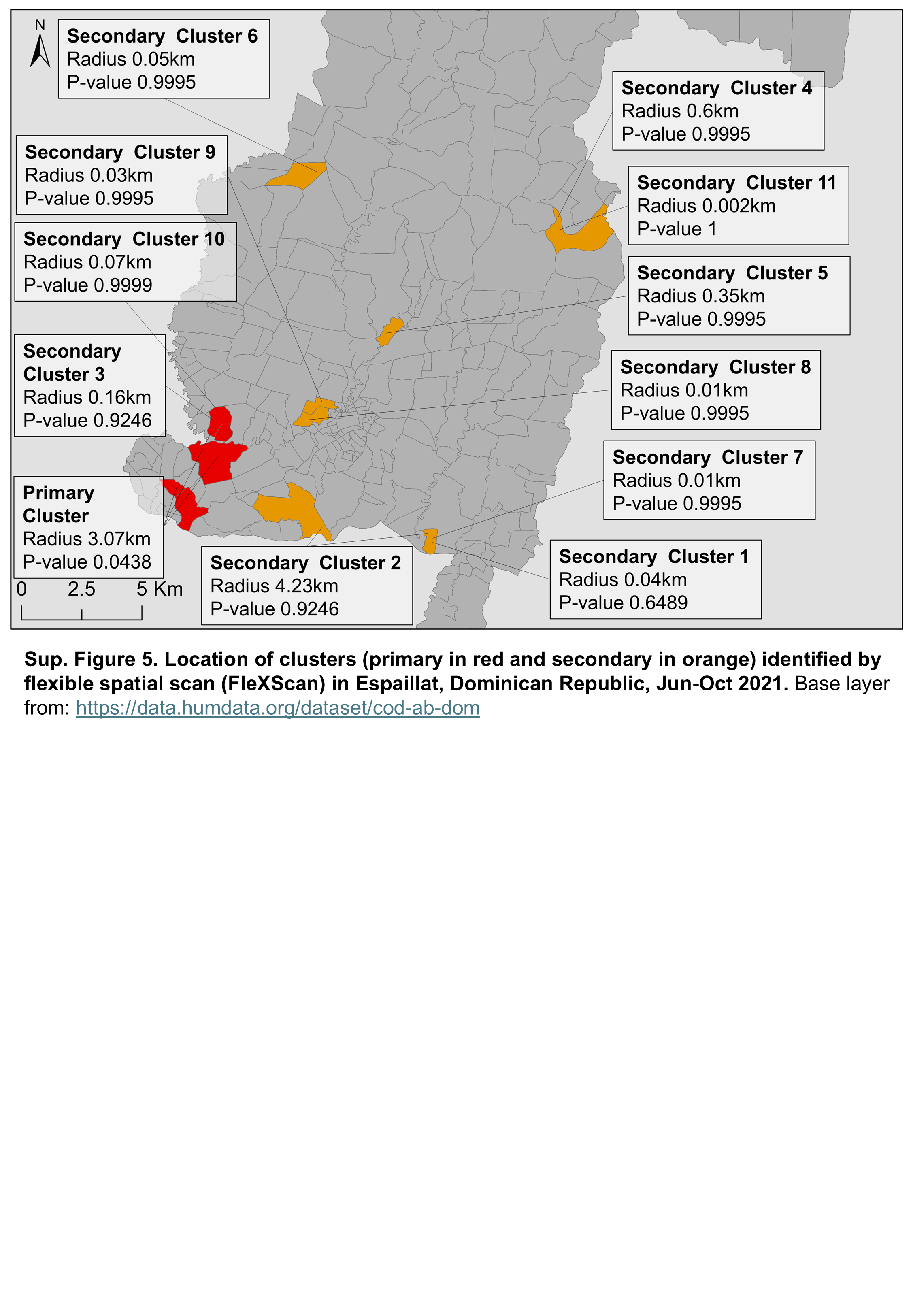

Supplement: S5 Fig — Base layer from: https://data.humdata.org/dataset/cod-ab-dom. (TIF) [file pntd.0013103.s008.tif]

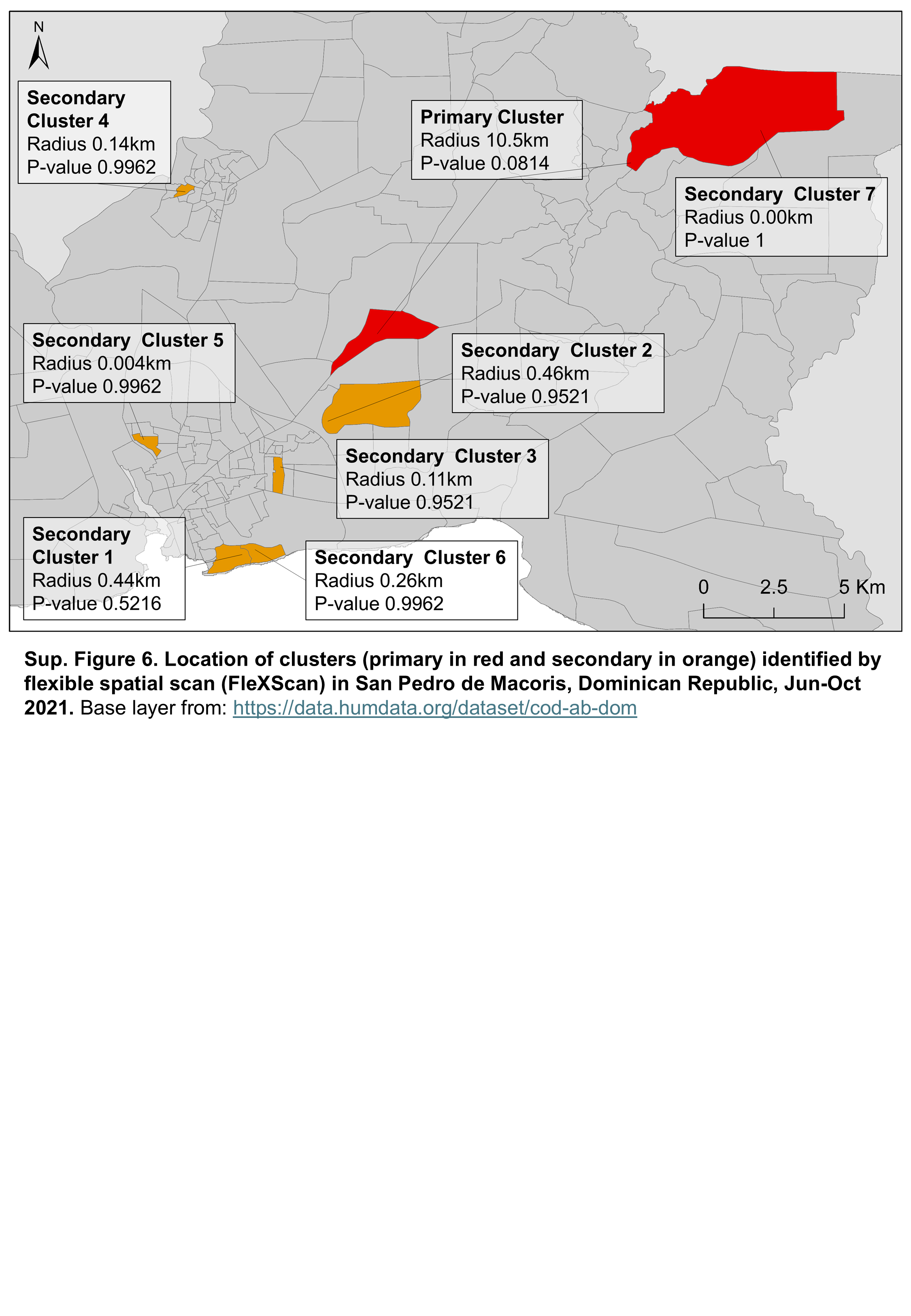

Supplement: S6 Fig — Base layer from: https://data.humdata.org/dataset/cod-ab-dom. (TIF) [file pntd.0013103.s009.tif]
