# Supplementary material for: Geospatial analysis of leptospirosis clusters and risk factors in two provinces of the Dominican Republic
Source: PLoS Negl Trop Dis. 2025 Jun 11;19(6):e0013103. doi: 10.1371/journal.pntd.0013103 (PMC12157080; doi:10.1371/journal.pntd.0013103)
Supplement: Attachment — Submitted filename: Response to reviewers_final.docx [file pntd.0013103.s011.docx]

**Reviewers' Comments:**

Dear editor,

We would like to thank you and the reviewer for your constructive and valuable feedback on our manuscript. We have carefully addressed all the comments to the best of our ability. Please find below our detailed responses, with references to the corresponding changes made in the manuscript.

**Reviewer #1:** The study focuses on Espaillat and SPM but **generalised the data to represent leptospirosis clusters for the entire country of Dominican Republic.** Would it be possible to include data from other areas of the country? As you rightly concluded, the drivers of leptospirosis transmission can be highly specific to the context of each province and as such, the characteristics/risk factors may very likely be different if other areas in the Dominican Republic were to be included in this study. Otherwise, I suggest **revising the title** to reflect the presented data.

**Authors**: We thank the reviewer for their time and careful review of our work. This analysis is nested within a larger research project entitled “Defining the pathogens, transmission characteristics, risk factors and geospatial hotspots of acute febrile infections (AFIs) in the Dominican Republic (DR). This research project had two arms, (i) a prospective AFI surveillance study conducted at two sub-national hospitals located in Espaillat and San Pedro de Macoris between 2019 and 2023, and (ii) a national, population-representative cross-sectional serological survey conducted between June and October 2021. Data analysed in our study was obtained on the national serosurvey, but only samples from the two provinces linked with the prospective AFI surveillance were tested for leptospirosis because of the substantial time and resources required to conduct microscopic agglutination tests. We have changed the title to “**Geospatial analysis of leptospirosis clusters and risk factors in two provinces of the Dominican Republic**”.

**Results:**

**Reviewer #1:** Were the participants healthy at the point of blood collection? From the writing, it is clear that this study was conceived as a result of the studies in Refs. 27 and 30, or maybe the other way around. Nonetheless, it would be helpful to the readers if there could be some form of linkage from the published papers to the present paper, so that we can understand the study design and not necessarily having to refer to the published papers.

**Authors**: Participants for the current study were recruited through a national population representative survey of general community members across the DR, i.e., they did not present with an acute febrile illness. The previous papers - references 27 (Nilles et al, 2023) and 30 (Nilles et al, 2022) – reported on SARS-CoV-2 for all participants in the national survey. Leptospirosis serology was only conducted for participants from two provinces. Testing for antibodies against leptospirosis was planned from the start of the national survey. However, conducting MAT is time-consuming and expensive, and therefore limited to participants in the two provinces that were included in a linked AFI surveillance study because of the possibility of conducting analyses that combined results from the serosurvey and AFI study. However, such comparisons are not the aim of the current study, thus only results from the serosurvey are presented in our manuscript. To clarify the relationship between the studies, we adjusted the reference to the AFI surveillance study (page 5, lines 121-123) and the survey design (page 6, lines 135-136):

“These two provinces were selected because they were included in a linked study on clinical surveillance of acute febrile illnesses (Supporting information).”

“The survey sampling design has already been described previously (29). In summary, a total of 10 communities (…)”

The information added to the Supporting information was:

“**Study design and participant selection**:

The analysis presented in this study was part of a larger research project entitled “Defining the pathogens, transmission characteristics, risk factors and geospatial hotspots of acute febrile infections (AFIs) in the Dominican Republic (DR). This research project had two arms, (i) a prospective AFI surveillance study conducted at two sub-national hospitals located in Espaillat and San Pedro de Macoris between 2019 and 2023, and (ii) a national, population-representative cross-sectional serological survey conducted between June and October 2021. In this study, we used data from the survey arm. The full description of the study design was previously reported by Nilles, et al, 2022 (1). Results for leptospirosis seroprevalence were available only for participants from the two provinces where the prospective AFI surveillance study was conducted. A full description of the surveillance study design can be found in Nilles et al, 2023 (2).”

**Conclusions:**

**Reviewer #1**: The main results described MAT findings of the seropositive participants in SPM and Espaillat. Additionally, the authors mentioned that the relationship between serogroups and reservoir mammals can be highly specific, and in turn, routes of human exposure are related to exposure to the reservoir mammals. I would expect the authors to discuss on the possible reservoir animals present in the hot spots or what could be other possible explanations for those serogroups to be present in the hot spots.

**Authors**: Thank you for your suggestion. We have expanded the discussion to include potential explanations regarding the reservoirs and serogroups identified in each province. The following changes were made (page 18. Lines 377-385):

“The relationship between serogroups and reservoir mammals can be highly specific, and in turn, routes of human exposure are related to exposure to the reservoir mammals. Thus, characteristics of clusters of high leptospirosis seroprevalence are likely to vary based on the predominant serogroup. In our analysis, the small sample size limited local cluster analysis based on the serogroup predominant in each province, which could have provided more comprehensive insights into specific risk factors and drivers of transmission. The main serogroups identified in Espaillat (results shown in the Supporting Information), were Australis, associated with wild and domestic animals, rats and mice, and Icterohaemorrhagie, mainly associated with rodents (54,55). In this province, clusters were not associated with self-reported rat exposure, yet several characteristics associated with clusters were indicative of remote impoverished, agricultural communities, which could be associated with the presence of reservoirs of Australis and Icterohaemorrhagie. In SPM, the main serovar identified was Australis, followed by Canicola, Djasiman, Icterohaemorrhagie and Pyrogenes equally distributed, which suggests a broader range of reservoirs driving transmission in the province.”

Reference added:

54.	Bharti AR, Nally JE, Ricaldi JN, Matthias MA, Diaz MM, Lovett MA, et al. Leptospirosis: a zoonotic disease of global importance. The Lancet Infectious Diseases. 2003;3(12):757-71.

55.	Petrakovsky J, Bianchi A, Fisun H, Nájera-Aguilar P, Pereira M. Animal Leptospirosis in Latin America and the Caribbean Countries: Reported Outbreaks and Literature Review (2002–2014). International Journal of Environmental Research and Public Health. 2014;11(10):10770-89.

**Summary and General Comments:**

**Reviewer #1:** The study presents insights into leptospirosis transmission in the Dominican Republic and employs a robust methodology. However, there are areas where the study could be improved.

**Authors:** Thank you for your comments. We made the suggested modifications to the manuscript.
